# Effect of seasonality, climatic and temporal factors on suicide attempts amongst patients from southern tunisia

**DOI:** 10.1192/j.eurpsy.2021.1566

**Published:** 2021-08-13

**Authors:** M. Tfifha, W. Abbes, W. Mahdhaoui, A. Kerkeni, M. Dhemaid, I. Rejeb, L. Ghanmi

**Affiliations:** 1 Department Of Psychiatry, regional hospital of gabes, gabes, Tunisia; 2 The Department Of Psychiatry, Hospital of gabes, Gabes, Tunisia; 3 Psychiatry, regional hospital of Gabes, Gabes, Tunisia; 4 Emergency Department, Hospital of gabes, Gabes, Tunisia

**Keywords:** Suicide prevention, temporal factors, climatic factors, Suicide attempts

## Abstract

**Introduction:**

Seasonal changes, climatic factors such as temperature, sunlight intensity and precipitations as well as temporal factors seem to have an influence on suicidal behavior.

**Objectives:**

Our study aimed to analyse the association between seasonal changes, climatic variations, temporal factors and suicide attempts.

**Methods:**

A retrospective descriptive and analytical study was undertaken including all patients consulting for the first time at Gabes psychiatry department from the 4^th^ March 2009 to the 25^th^ September 2020 for suicidal attempt. Sociodemographic and clinical data as well as suicidal attempts’ characteristics were assessed. Meteorologic data, related to the years 2009 through 2020, were obtained from the official weather website of Tunisia.

**Results:**

278 patients were collected, including 217 female. Mean age was 26. Suicidal patients were unmarried (75.9%), childless (79.1%) and unemployed (47.5%). Results showed that suicidal attempts occurred most frequently in summer (32.5%) specifically in June and July (10.9% for each). Regarding the distribution of suicide attempts over the days of the week, the highest rate was observed on Monday (22.5%) and the lower one on Friday. There were a correlation between high temperature and suicide attempt by hanging (p=0.006), between days of sunlight and manifestations preceding the suicidal attempt (p=0.04) and between rainfall and anxiety disorder leading to suicidal attempt (p=0.03).We finded also an association between the summer and risk behavior such as runaways (p=0.024).

**Conclusions:**

A better identification of seasonality, climatic and temporal factors in suicidal behavior could allow a better prevention in suicidal attempts and a reduction in death by suicide

